# International Validity of the Athlete Psychological Strain Questionnaire (APSQ): A Scoping Review

**DOI:** 10.3390/diagnostics16030486

**Published:** 2026-02-05

**Authors:** Teodora-Simina Dragoiu, Florentina Ligia Furtunescu, Adela Caramoci, Oliver R. Runswick

**Affiliations:** 1 Department of Public Health and Management, Carol Davila University of Medicine and Pharmacy, 050463 Bucharest, Romania; 2 Department of Sports Medicine, Carol Davila University of Medicine and Pharmacy, 050474 Bucharest, Romania; adela.caramoci@umfcd.ro; 3 Department of Psychology, Institute of Psychiatry, Psychology and Neuroscience, King’s College, London SE5 8AB, UK; oliver.runswick@kcl.ac.uk

**Keywords:** athlete psychological strain questionnaire, sport mental health assessment tool-1, mental health, performance psychology, screening, cross-cultural validity

## Abstract

**Background/Objectives:** Mental health screening in athletes is an essential process to support well-being and sustainable performance. The Athlete Psychological Strain Questionnaire (APSQ) represents the ten-item triage step of the Sport Mental Health Assessment Tool-1 (SMHAT-1), created by the International Olympic Committee. We aimed to gather relevant information concerning the validity of the APSQ in different cultural settings. **Methods:** The study was designed as a scoping review and included 19 articles from Scopus, PubMed, Embase, Web of Science, and Google Scholar databases. Articles were written in English and tested the APSQ validity. **Results:** Different studies used the original or the translated version of APSQ and tested its benchmarked validity against other validated questionnaires, ran confirmatory and exploratory analyses, test–retest stability, calculated diagnostic metrics, and internal consistency. Most studies agreed on the good internal consistency, with optimal Cronbach’s alpha values, test–retest reliability, three-factor solution, convergent validity with scales assessing distress, divergent validity with well-being scales as demonstrated by significant correlation coefficients. The cut-off showed good accuracy for anxiety and depressive symptoms in terms of AUC, sensitivity, and specificity, but, in some cases, a limited ability (based on the AUC) to detect sleep concerns, alcohol misuse, substance use, and disordered eating (as measured by BEDA-Q). Some authors suggested that using different cut-offs, including all questionnaires from SMHAT-1 Step 2, or using a clinical interview, might mitigate these concerns. **Conclusions:** Different cultural environments might influence the validity of APSQ. A structured translation and validation study is advised before implementing APSQ in a different language.

## 1. Introduction

The athletic population faces specific stressors that are not seen in the general population, such as high expectancy for performance, rigorous lifestyle rules, increased physical demands, proneness to serious injuries, or transition to a different career [1,2,3,4,5,6]. In this context, the prevalence of common mental health symptoms in athletes is comparable to the general population, but the evidence presents a wide range of estimates [1,2,7,8,9,10,11,12,13]. Studies reporting on the prevalence of common mental health problems in athletes have identified different factors that may affect prevalence, such as gender, where female athletes are found to have a greater susceptibility to psychological distress/depression/eating disorders, [11,14,15,16] or the type of sport where participation in team sports has been associated with a lower prevalence of depression and psychological distress [10,11,12], but higher prevalence of alcohol misuse compared with individual sports [15]. There is a need for sports medicine practitioners, psychiatrists, and psychologists to have specific screening tools that can be used to monitor the health of athletes while accounting for these unique stressors [5,11,16,17,18]. Tools such as the Athlete Psychological Strain Questionnaire (APSQ) have been developed by international governing bodies, but there is limited understanding of their validity across cultures [16,19,20].

Research in sports medicine traditionally focused on the prevention and treatment of physical impairments rather than psychological ones [1,11]. In light of rising awareness of the importance of mental health and its impact on the quality of life and success, the fields of sports psychiatry and psychology have developed and represent essential components of elite athletes’ healthcare [21]. To maintain and increase performance, targeted interventions to safeguard and optimise the mental health of elite athletes need to be implemented [1,4,5,20]. Athletes tend to hide mental health struggles until their coping strategies are exhausted, and symptoms of psychological strain emerge [1,17,22]. Some may experience somatic symptoms or engage in harmful external coping practices, such as increased alcohol consumption [1,23]. Therefore, there is a need for valid screening tools to support healthcare providers in understanding when athletes may need preventative support or treatment. 

Mental health screening questionnaires for athletes have been developed, but there are no international guidelines or standardised protocols to establish a gold-standard tool [4,18,20]. According to the concept of screening, these questionnaires are meant to identify individuals at risk and not to provide a diagnosis [4,18,20,24]. Self-assessment questionnaires rely on the mental health literacy of the responders and their willingness to reveal certain symptoms [18,20]. The most commonly used mental health screening questionnaires are the Patient Health Questionnaire-9 for depression (PHQ-9) [25], the Generalised Anxiety Disorder Scale (GAD-7) [26], the Alcohol Use Disorders Test (AUDIT) [27], the General Health Questionnaire (GHQ-28) [28], the Yale–Brown Obsessive–Compulsive Scale (YBOCS) [29] and so on. However, these tools often do not account for the specific stressors faced and the unique contexts that elite athletes live within. 

Tailored questionnaires relevant to the athletic population have been developed, such as the APSQ [24], the Baron Depression Screener for Athletes (BDSA) [16,30], and the Athlete Sleep Satisfaction Questionnaire (ASSQ) [31]. The most popular questionnaires that assess the general mental health dimension and not only a targeted problem are the Athlete Psychological Strain Questionnaire (APSQ), the Sport Interference Checklist [32], the Student Athlete Relationship Inventory, and the Mental Health Disorders Screening Instrument for Athletes [18]. To address the complex picture of screening options and provide athlete support teams with a single specific tool, in 2020, the Mental Health Working Group (MHWG), part of the International Olympic Committee (IOC) introduced the Sport Mental Health Assessment Tool (SMHAT-1) and the Sport Mental Health Recognition Tool (SMHRT-1) as mental health screening tools designed for athletes [4,12,20,24]. The SMHAT-1 can be used by sports medicine doctors with or without the assistance of a mental health professional [4,12,20,24]. The SMHRT-1 can be utilised by non-professional members of the team/family and can raise the suspicion of a mental health concern [4,12,20,24]. The SMHAT-1 includes a three-step approach [4,12,20,24]. The first step uses the APSQ as a triage tool and for the assessment of psychological strain [4,12,20,24]. If the score of APSQ is more than or equal to 17, an additional set of questionnaires that targets specific conditions is performed (step two) [4,12,20,24]. Step three includes additional questionnaires that assess for less common mental health conditions (such as bipolar disorder or psychotic symptoms), along with a brief intervention and monitoring plan or the recommendation for a formal clinical assessment to diagnose and treat the potential mental health problem [4,12,20,24]. As a tool that combines many of the previously developed options and is recommended by the IOC, the APSQ will be the focus of this review. 

Rice et al. created the APSQ using a multistage process [1]. The questions were developed after an exhaustive review of the athletes’ mental health literature, with the lead author creating the initial 12 items in the questionnaire and two other authors reviewing the items [1]. The final version consisted of 10 questions with response options ranging from 1 point meaning ‘none of the time’ to 5 points meaning ‘all of the time’ [1]. This original APSQ version included a three-factor structure: External Coping (two items), Self-Regulation (four items), and Performance (four items) [1,3]. The original three-factor structure of APSQ resulted from exploratory analysis in a calibration sample (497 participants) and was validated by confirmatory factor analysis in a validation sample (510 participants) [1]. The questionnaire was tested in a cross-sectional study that involved 1007 elite, male professional Australian athletes, where results confirmed that the APSQ was reliable in correctly identifying athletes with significant psychological distress and therefore an efficient screening tool for athletes [1]. As a tool recommended by the IOC, its use spans many countries and cultures, and there is a need to understand its validity in a wider range of samples [3,6,7,33,34,35,36,37]. 

The validity of the SMHAT-1 questionnaires has been assessed in a range of studies [8,14,15,24,38]. However, since the initial step (the APSQ) of SMHAT-1 decides the need for further evaluation, it is of paramount importance to have a high sensitivity/a low rate of false negative results. Otherwise, athletes who might benefit from mental health support are neglected. Even if the questionnaires from steps 2 and 3 are highly performant in identifying mental health problems, they depend on the validity of APSQ. Therefore, the purpose of this scoping review was to collate evidence on the effectiveness of APSQ in identifying athletes in need of clinical evaluation and mental health support across different samples and populations, including the validity of APSQ in different languages [8,14,15,24,38].

## 2. Materials and Methods

The method was developed in line with the framework suggested by Arksey et al. [39] and the Joanna Briggs Institute Reviewers’ Manual recommendations [40]. We followed the PRISMA extension for scoping review checklist and guidelines [41]. The PRISMA extension for scoping review checklist was included in the Supplementary File S1 [41]. 

### 2.1. Identifying the Research Question

The research question sought to be comprehensive enough to cover all the literature available on this topic without adding irrelevant articles or an unmanageable number of articles. Following the ‘Population, Context and Concept’ structure [40], the research question was ‘What evidence is available to determine if the Athlete Psychological Strain Questionnaire is a valid mental health screening tool worldwide?’.

### 2.2. Identifying Relevant Studies

To identify relevant studies, we investigated multiple databases, including PubMed, Embase, Web of Science Core Collection, Scopus, and Google Scholar, to ensure a thorough search. The inclusion criteria were articles written in English that investigated the effectiveness of the APSQ as a screening tool for the mental health of athletes, regardless of the athletes’ performance level, the study type, or the publication date. The terminology ‘valid’ encompasses all the relevant statistical processes required for the validation of questionnaires, including reliability assessment. The exclusion criteria were articles that did not test the APSQ’s validity, articles written in other languages, book chapters, pre-print manuscripts, dissertations, conference presentations, or unpublished data. Due to the questionnaire design, meant for athletes over 16 years old, we checked if all the participants included in the study met this requirement.

### 2.3. Study Selection

Generation of search terms utilised the PICO structure, focusing on the athletic populations, use of the APSQ as an intervention, and validity and reliability as outcomes. We did not specify comparison due to a wish to explore which other measures were used to establish validity in the results of the search. The final keyword structure used was:

(Athlete OR athlete* OR professional athlete* OR elite athlete* OR collegiate athlete* OR sport*)

AND

(Athlete Psychological Strain Questionnaire OR APSQ OR SMHAT OR Sport Mental Health Assessment Tool 1)

AND

(valid* OR reliability OR reliab* OR psychometric* OR psychometric properties OR properties)

The keywords were not restricted to a specific section. We did not use any filter/automatic exclusion tools to narrow our search. The search was conducted on PubMed, Embase, Scopus, and Web of Science on 2 December 2024. Articles were collected in Rayyan in order to ensure access to all the authors involved and to easily remove duplicates. An updated literature search was performed in May 2025, identifying one additional article for inclusion and a second update in November 2025 (PubMed = 71 results; Web of Science = 124 results; and Scopus = 472 results), leading to four new studies being incorporated into the final analysis.

Each article was first screened by title or abstract. The ones that we deemed eligible were sought for retrieval and analysed for inclusion criteria. We looked for the risk of bias that was usually described in the ‘limitations’ section. Some of the articles used the STROBE guidelines for cross-sectional studies. 

**Figure 1:**
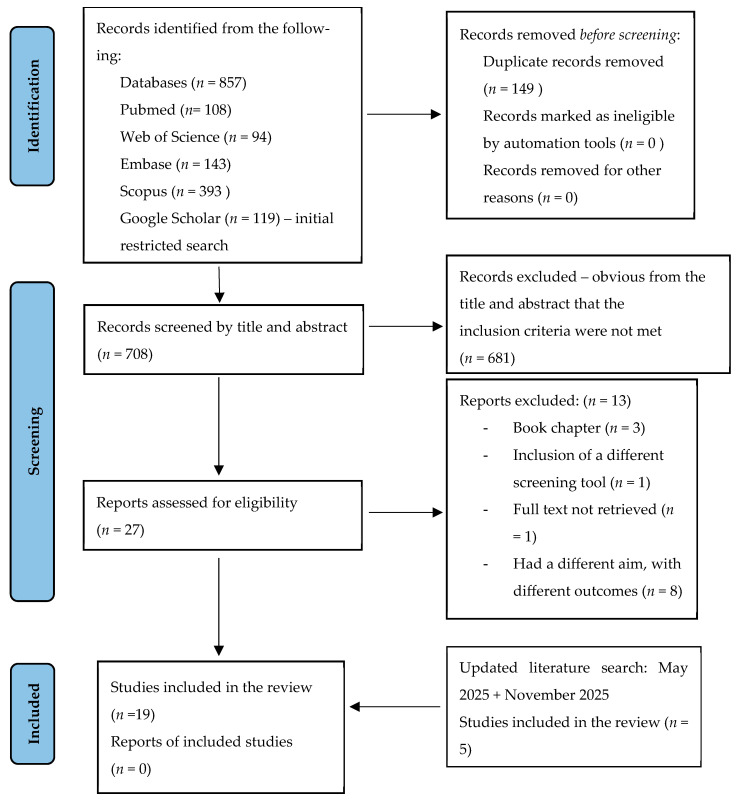
The data collection process. PRISMA Flow Diagram. Source: Page MJ et al. BMJ 2021;372:n71. doi: 10.1136/bmj.n71 [42]. This work is licenced under CC BY 4.0. To view a copy of this licence, visit https://creativecommons.org/licenses/by/4.0/ (accessed on 15 May 2024).

All authors were involved in the creation of the study design, but one author was responsible for drafting it. After being reviewed by all the authors, the same author made all the adjustments. In terms of selection bias, we applied a double screening method for a single database. Two authors were involved in the initial selection process of articles from PubMed, and there were no disagreements. One author carried out the rest of the selection process. If the articles were not available in open access, the full texts were retrieved using the University’s library platform. One article was still not available as full-text and, therefore, not included. Figure 1 includes the PRISMA Flow Diagram [42].

### 2.4. Charting the Data

One author was responsible for collecting the targeted data in an Excel document. Three spreadsheets were created: one including the studies’ characteristics, one with details about the translation process, and another one with results from the validation process. The study characteristics comprised the following aspects: the type of study, the number of participants, the characteristics of the sample, the aim of the study, and the limitations. The spreadsheet with details about the translation process included the language of translation and the translation process. The ‘validation results’ spreadsheet included the expected parameters described in validation studies: exploratory and confirmatory factor analysis, convergent and divergent validity, internal consistency, test–retest reliability, false negative rate, false positive rate, and cut-off scores.

### 2.5. Collating, Summarising, and Reporting the Results

The APSQ test was written in English, as this is a global language. However, to become the gold-standard triage tool in a certain country, the questionnaire requires translation into the country’s official language. We explored which countries have published the translated version of APSQ and how they validated it. As mentioned in the ‘Introduction’, the study’s aim was to assemble all available data on the validity of APSQ. Different validation studies might include other statistical evidence, so we structured the results from each study in separate essential components (described in the ‘validation results’ spreadsheet). The translation process was discussed separately for each individual study. Characteristics of the selected studies were described in the second subchapter of the ‘Results’ section.

## 3. Results

### 3.1. Translation into Different Languages

Most of the translation and validation research has been carried out in Asia and Europe. The currently translated and validated versions of APSQ are the Arabic, Japanese, Persian, Turkish, Croatian, Polish, Indonesian, and Spanish versions [3,7,19,36,37,43,44]. Other translations are listed on the IOC website, yet corresponding validation studies were not retrieved by our search [45]. 

Each study followed a structured translation process that included multiple steps: translation from English into the targeted language; analysis of the translated version by experts in sports science, language experts or athletes; back translation into English by bilingual experts; comparison of the original English version with the one that resulted from back translation; review by experts and final amendments; and pretesting for comprehension and relevance in a small sample [3,7,19,36,37]. The studies generally adhered to this framework but did not always include all the steps. 

We summarised the translation process for each study in Table 1.

### 3.2. Characteristics of the Included Studies

The studies included in the review were designed as cross-sectional studies. The majority of the studies included either solely male athletes [1,2,3,15,38] or predominantly male athletes [9,19,37]. The studies explored the validity of APSQ/the translated version of APSQ, using specific statistical analysis. The study design, the number of participants, the characteristics of the sample, the main aim of the study, and the limitations were summarised in Supplementary Material Table S1. Although most studies included elite-level athletes [2,7,8,14,24,36,46], some involved college athletes [15,47,48,49], non-elite athletes [9], or both elite and sub-elite athletes [19,37,43]. A critical appraisal of these studies was performed after their inclusion, using the Joanna Briggs Institute checklist, and is presented in Supplementary Material Table S2 [50,51]. It is worth noting that not all criteria were relevant to validation studies.

### 3.3. Validation Analysis

Each study performed a specific statistical analysis for the validation process. We have reported each parameter separately. 

#### 3.3.1. Exploratory and Confirmatory Factor Analysis 

Both exploratory and confirmatory analyses were performed to identify the factor structure of the English/translated version of APSQ [7,8,9,19,36]. The Kaiser–Meyer–Olkin (KMO) test and Bartlett’s test of sphericity showed adequacy for factor analysis, a significant correlation between items, and relevance for factor reduction [1,36]. The Japanese version of APSQ supported the one-domain structure, with adequate values of the comparative fit index, root mean square error of approximation, and Chi-square statistics in the confirmatory factor analysis, after covariance adjustments [3]. Additionally, the one-domain structure showed good factor loadings and appropriate model fit indices values in a study meant to validate the English version of APSQ in a non-elite sample [9]. In contrast, the two-factor structure of the Croatian APSQ was confirmed using the Cattell scree test and varimax rotation: External Stressors (questions six and seven) and Internal Psychological Strain (the rest of the questions) [36]. Despite those inconsistencies, most studies supported the three-domain structure of APSQ—Self-Regulation, Performance, and External Coping—proving adequate model fit indices [7,9,19,44,47], in some cases, after adjusting for two-error covariance [8]. The Indonesian APSQ version showed adequate model fit for both the two- and three-factor solutions [44].

#### 3.3.2. Convergent and Divergent Validity 

Significant convergent validity between the K-10 or K-6 and all the domains of APSQ was demonstrated in multiple studies [1,2,3,7,19,24]. Fair-to-moderate convergent validity was detected between the Spanish APSQ and K-10 [43]. Convergent validity was also identified between the Turkish and Spanish versions of APSQ and the DASS-21 [7,43]. Furthermore, significant positive correlations were determined between the APSQ and Generalised Anxiety Disorder questionnaire (GAD-7) and Patient Health Questionnaire—8 (PHQ-8), which had been validated as screening tools for dysfunctional anxiety and detection of depressive symptoms [9,26,52]. In addition, a study involving collegiate athletes showed convergent validity between the domains of the APSQ with either the Athlete Mental Health Belief and Help Seeking Attitude Scale or the Attitudes Toward Seeking Professional Psychological Help Short Form [47].

Significant divergent validity between APSQ and the Warwick–Edinburgh Mental Well-Being Scale (WEMBS) was pinpointed in some studies [1,2]. In addition, significant negative correlations were found between APSQ and other validated questionnaires which reflect psychological well-being, such as the World Health Organisation (WHO) Well-Being Index 5, the Ryff Mental Well-Being Scale (RMWBS), and the Mental Health Continuum—Short Form (MHC-SF) [3,9,19]. Moreover, the Indonesian APSQ demonstrated significant divergent validity with the Mental Toughness Index [44].

#### 3.3.3. Reliability Assessment

The internal consistency assessment of the APSQ was analysed for the Arab, the Japanese, the Turkish, the Persian, the Croatian, the Spanish, the Indonesian, and the original versions of APSQ, using Cronbach’s alpha value [3,7,9,19,36,37,38,43,44,47,53]. Additionally, test–retest stability was tested in other studies [7,9,37]. 

Cronbach’s alpha values varied across the studies, demonstrating good internal consistency in some studies [3,7,15,43,48] and adequate internal consistency (Cronbach’s alpha above 0.70 but below 0.80) in others [19,36,37].

The test–retest reliability yielded excellent ICC values for the Arabic version [37], good values for the Turkish version [7], and acceptable values for the Spanish version [43]. In a study involving non-elite athletes, questions 9 and 10 showed good ICC values, compared to questions 1 and 3, which demonstrated poor reliability after one month [9]. 

Separate Cronbach’s alpha values for each domain of the APSQ were calculated in the studies involving Turkish, Croatian, and Indonesian collegiate athletes and amateur athletes [7,9,36,44,47]. The External Coping domain showed either higher Cronbach’s alpha values [36] or lower values compared to the rest of the domains [7,44]. In a different study, the highest value was observed for the Self-Regulation domain [9]. Internal consistency was either higher [24,48] in the female sample or lower compared to male athletes [2].

#### 3.3.4. Performance Metrics

Diagnostic metrics of the APSQ 17 cut-off score for common mental health issues were mainly analysed using the screening tests included in the SMHAT’s second step [2,24,38,48,49]. In the study testing the Polish APSQ validity, the authors also used a brief clinical interview for this analysis [8]. The main findings are summarised in Table 2.

Anderson et al. highlighted that female athletes had lower rates of false negative results on APSQ, apart from the PHQ-9, where male athletes did not have any false negative results [14], and that Olympic athletes had higher false negative results of APSQ than Paralympic athletes [14]. Considering the significant rate of false negative results, especially for common mental health conditions, the authors of the study suggested that a better screening protocol would be to include the additional questionnaires from SMHAT-1’s step two, together with APSQ, or at least include a question related to suicidal thoughts [14]. 

#### 3.3.5. Cut-Off Scores

Additional threshold scores, including a moderate (≥15) category, a high (≥17) category, and a very high (≥20) category, were described by the creators of this tool [2]. All three cut-off scores of APSQ: 15, 17 and 20 (moderate, high, and very high psychological distress) had good area under the curve (AUC) values when compared to K-10, meaning they are reliable in correctly identifying levels of psychological strain [2]. The cut-off value of 20 presented the highest discrimination ability in this study, considering the specificity, sensitivity, and the AUC values [2]. Another study that benchmarked the three cut-off values against different levels of distress on K-10 was the one that validated the Persian version of the APSQ [19]. The very high cut-off value (≥20) had the highest Youden’s index and the best ability to identify those who truly have a problem [19].

Similarly to the aforementioned data, other studies have shown that a cut-off score of ≥21 or ≥23 would have a good balance between sensitivity and specificity [8]. In a study that compared APSQ against a clinical interview, the value of ≥25 had adequate sensitivity, specificity, positive predictive value, and negative predictive value, as well as the highest Youden index [8]. Still, the authors suggested that a cut-off value of ≥23 would be optimal and avoid a high incidence of false positive results while minimising the risk of overlooking true positive cases [8]. Lower cut-off values, such as 10 or 14, ensured that no cases were missed out, but had a very high prevalence of false positive results [8]. The study also revealed that even a brief clinical interview by a mental health specialist might help distinguish those who are truly in need of support compared to those who experience normal short periods of increased psychological stress [8]. The 23 cut-off score was also deemed to be optimal in terms of sensitivity, specificity, and AUC of the ROC, in a study including non-elite athletes [9].

## 4. Discussion

This review aimed to explore whether APSQ is a valid, reliable screening tool worldwide. We identified validation studies in different cultural settings and gathered compelling evidence on APSQ’s performance and limitations. We briefly assembled and summarised the main results of these studies and further discussed the implications of these findings.

The majority of the studies showed that APSQ is a reliable, valid psychometric tool that can be used as a triage tool in athletes’ mental health assessments in different languages [7,8,9,19,36,43]. There was a general agreement on the good internal consistency of APSQ [2,7,15,24,36,48]. However, not all studies reported Cronbach’s alpha values above 0.80 [19,36,37]. Although all reported values exceeded 0.70 [19,36,37], moderate internal consistency does not provide the same level of confidence for practical use. The test–retest reliability was assessed after one week, two weeks, or within one month, and the general APSQ score showed adequate reliability over time [7,37]. This showed that APSQ is reliable over time and that the questions have strong intercorrelations. However, the Spanish APSQ version did not prove to have absolute reliability, and the authors concluded that it should be used cautiously when multiple re-testing over time is required [43]. Not all studies conducted a second measurement to explore stability over time, and it is worth mentioning that all included studies were cross-sectional in design, which inherently entails certain limitations.

As a triage instrument, we considered the AUC and the FNR values to be particularly relevant for assessing diagnostic performance. There was a consensus regarding the diagnostic accuracy of the APSQ for anxiety, depression, and self-harm thoughts [8,14,38,46,48,49]. In addition, most studies showed poor diagnostic performance for alcohol misuse, sleep disturbances, and disordered eating (as measured by BEDA-Q) [8,14,46,49]. However, APSQ had optimal performance for measuring disordered eating when benchmarked against the EDE-QS [49]. Regarding the APSQ performance to detect substance use, most studies highlighted a limited accuracy [14,46,49], though there was one study with no missed cases (negative APSQ and a positive CAGE-AID) [24] and one showing an acceptable AUC value, but a high FPR [8]. Despite the low false negative rates for GAD-7 and PHQ-9 compared to the rest of the mental health concerns, there were authors who highlighted that these were actually significant in clinical practice [14]. The general false negative rate was deemed to be high for a triage tool by some authors [8,14,48]. Women tended to have a lower false negative rate, and men a lower false positive rate [8,14]. Regarding false positive rates, some studies showed relatively high rates for CAGE-AID [46] and question 9 of the PHQ-9 [46,49]. 

Being a self-reported tool, APSQ is affected by the athlete’s ability to understand the questions and the confidentiality attached to these kinds of studies, the athlete’s willingness to accurately respond to the questions, without randomly selecting an answer, and the athlete’s belief that this will help research in mental health and therefore contribute to better mental health support for athletes [7,36,48]. There might also be differences across genders, as female athletes generally tend to disclose their problems more than males (thus the lower false negative rate of APSQ in women) [54,55]. Secondly, some of the screening tests from Step 2 have not been tested on athletes [24]. In addition, part of the false negative/positive results might have also been influenced by the high prevalence of mental health problems in the athletic population or in the female gender, such as eating disorders [11,16,55].

An alternative cut-off value of >20 (very high psychological distress), considering sensitivity/specificity/Youden index, was mentioned in some studies [1,2,19]. In other studies, the cut-off value of 23 had the most appropriate indicators [8,9]. The need for further validation of cut-off scores in order to have an adequate sensitivity and specificity of APSQ was mentioned in a few studies [3,8,48]. These studies suggest that the threshold value might not be accurate worldwide, due to cultural influences and mental health preconceptions [2,9,24]. Taking into account that APSQ is a triage tool, a lower cut-off value would ensure that fewer athletes in need of mental health support are missed. The differences in cut-off values performance might have been influenced again by cultural determinants or by a particularly high incidence of mental health problems in certain regions. Further studies need to benchmark the APSQ sensitivity against a clinical interview and set up cut-off scores that have an acceptable Youden’s index for a triage tool. This might be different according to the cultural setting.

Some authors suggested that better triage options might be to use other validated tests (such as K-10), to include the SMHAT-1 step 2 battery of tests, to start directly with the questionnaires from step 2, or to perform a short clinical interview [8,14,48]. In a clinical setting, the mental health specialist can build up the confidence required for such confessions, which is not always the case when using a questionnaire. Therefore, a clinical interview might have a higher accuracy in detecting mental health problems. Considering that mental health specialists and sports medicine consultants have limited time available, some suggestions might not be feasible. Attention should also be given to athletes’ acceptance of a time-consuming mental health screening test [49]. Additional research should investigate both the athletes’ and the physicians’/mental health practitioners’ acceptability of APSQ as a mental health screening tool.

Most of the studies showcased that the three-domain structure (External Coping, Performance, and Self-Regulation) was statistically valid [1,7,8,9,19]. The differences in the factor structure might again be attributed to cultural influences, inaccurate responses, or unwillingness to disclose mental health problems. This is particularly important as it might reflect the utility of using a self-assessment screening tool. If that specific population does not normally disclose mental health problems via this sort of screening, the purpose of the questionnaire is not attained. Moreover, variability in the factorial structure, together with the lack of formal assessment of cross-cultural influences, might affect the generalisability of score interpretation and the ability to compare scores across cultural contexts.

All the translated versions of APSQ that were discussed followed a structured, rigorous process and proved to be reliable and valid. They were tested for convergent and divergent validity with validated, commonly used tests. The results demonstrated that the APSQ performed similarly if benchmarked against such tests [1,2,3,7,9,19,24]. Despite the rigorous translation process, there might have been elements of this process that are difficult to replicate or assessments that are subjective. Therefore, there might have been differences or particularities in each of these translation processes.

Part of the included studies recruited exclusively or predominantly male athletes [1,3,37,38] or athletes from a specific performance level (amateur/collegiate) [9,47,48], which may limit the generalisability of the results to the wider athletic population.

As with any other study, this review needs to be considered in light of its limitations. First, the quality assessment of the included papers comprised criteria that were not fully applicable to validation studies. However, we did assess the steps performed in the translation process to better understand its quality. A systematic review might further address this aspect, though the data available is not comprehensive enough to pursue this kind of study at this point. A more in-depth analysis of the factors that might have influenced the results of these studies would be beneficial. Lastly, we did not include papers from the grey literature. The results of this review highlight again the importance of considering the various cultural differences when validating or developing a mental health screening tool, and further investigation will be required to build on this initial review.

## 5. Conclusions

This scoping review aimed to explore the use and validity of APSQ across cultures and languages. The main findings of the review encourage the usage of APSQ as a triage tool, with the caveats that it needs further validation across different countries (with specific cut-off values testing) and the potential addition of items (such as questions testing for eating disorders). These findings are highly relevant for clinicians who perform mental health screening. Practical solutions to address diagnostic performance limitations for certain mental health conditions, as proposed by authors of the included studies and summarised in the Discussion section, could be adapted according to specific circumstances. While there is no gold standard for the mental health assessment of elite athletes, to ensure equal chances in competitions, reliable tools and management protocols are desirable.

## 6. Future Directions

To the knowledge of the authors, there is limited research available concerning the mental health of Paralympic athletes who might face additional or different challenges, such as classification and the nature of their impairment. These stressors might influence the APSQ validity; therefore, studies that address this context are important. In addition, future studies might focus on validating the translated versions of APSQ that have not yet been validated in specific cultural settings, along with cross-cultural measurement invariance testing.

## Figures and Tables

**Table 1:** APSQ translation process in different languages.

References	Language	Translation Process
Alhowimel AS, Alenazi AM, Alshehri MM, Alqahtani BA, Aljaman A, Alzahrani H, et al. “Translation and Validation of the Arabic Version of the Athlete Psychological Strain Questionnaire”. *J Sport Rehabil*. 2023 Aug 1. 32(6):709–12 [37]	Arabic	- Translation - Review by experts - Back translation - Pretesting—21 athletes - Final editing—no alterations after the pretest
Ojio Y, Matsunaga A, Kawamura S, Horiguchi M, Yoshitani G, Hatakeyama K, et al. “Validating a Japanese Version of the Athlete Psychological Strain Questionnaire”. *Sports Med Open*. 2021 Dec [3]	Japanese	- Initial translation - Review by 2 professional athletes - Back translation - Final approval
Lima Y, Atatürk Şehr Hastanes B, Hekmlğ Bölümü S, Denz Öz N, Denerel N, Özkaya Ö, et al. “Validity and reliability of the Turkish version of Athlete Psychological Strain Questionnaire (APSQ)”. *Spor Hekimliği Dergisi*. 2022 Mar 4; 57(3):147–54 [7]	Turkish	- Translation—language and sports science experts - Reverse translation (Turkish–English) - Comparison with the original English version - Analysis, final amendments, confirmation of the final version—sports science experts - Content validity index—assessed by three experts in sports science—0.90 (appropriate validity)
Azadi H, Meshkati Z, Rice S. The effects of training conditions on athletes’ mental health throughout the COVID-19 pandemic: “Psychometric validation of the Persian athlete psychological strain questionnaire”. *Apunts Sports Medicine*. 2024 Apr 1; 59(222):100437 [19]	Persian	- Translation—2 bilingual researchers, independently, experts in this field - Comparison between the 2 Persian versions - Optimisation of the Persian translation - Back translation—English native speaker - Comparison with the original English version - Further amendments to the final Persian version - In-person, cognitive interview to assess the comprehension and clarity of the questions
Sore K, Franic F, Androja L, Batarelo Kokic I, Marčinko D, Drmic S, et al. “Translation, Cross-Cultural Adaptation, and Validation of the Croatian Version of the Athlete Psychological Strain Questionnaire (APSQ)”. *Sports 2024*, Vol 12, Page 228. 2024 Aug 22; 12(8):228 [36]	Croatian	- Translation to Croatian by a bilingual researcher - Evaluation by a Croatian professor—2 minor alterations - Back-translation by an English interpreter proficient in Croatian - Comparison with the original English version by the bilingual research team—no changes - Pilot study—20 athletes—additional amendments
Waleriańczyk W, Krzywański J, Gorgol J, Konopka K, Kuśmierczyk A, Lisek G, et al. “Diagnostic effectiveness of the Sport Mental Health Assessment Tool 1 supplemented with a brief clinical intake interview in a cohort of Polish elite Olympic athletes”. *Br J Sports Med* [Internet]. 2024 Oct 23; bjsports-2024-108919 [8]	Polish	- Translation by two researchers - Reverse translation by two researchers - Pretesting for comprehension—10 athletes
García-Rubio J, González-Devesa D, Diz-Gómez JC, Carlos AP. “Validity and Reliability of the Spanish Version of the Athlete Psychological Strain Questionnaire”. *J Sport Rehabil.* 2025 Jan 31: 1–6. doi: 10.1123/jsr.2024-0284. Epub ahead of print. PMID: 39889693 [43]	Spanish	- Forward translation by a native English speaker, proficient in Spanish - Review by a committee - Reverse translation by a native Spanish speaker, proficient in English - Pretesting - Revised final version
Putra, M.F.P., Rahayuni, K., Wanena, T., Larung, E.Y.P., Sinaga, E., Zainuri, A., Numberi, G.K.I., Hidayat, R.R., Wandik, Y., Togodly, A. (2025). “Adaptation and Psychometric Validation of the Indonesian Version of the Athlete Psychological Strain Questionnaire”. *Sport i Turystyka*. Środkowoeuropejskie Czasopismo Naukowe, 8(3), 157–176 [44]	Indonesian	- Forward translation by 2 English experts - Revision of the translation and convergent validity analysis with a different tool by 3 sports psychology experts - Revision by an Indonesian expert - Pretesting of the other tool on 9 athletes - Reverse translation of the Indonesian APSQ by English experts - Review of the original vs. the one resulting from reverse translation by the creator of the APSQ - Lastest version of the Indonesian APSQ

**Table 2:** Diagnostic performance of the established cut-off for different mental health concerns.

Mental Health Concern	Diagnostic Metrics
Anxiety (as measured by GAD-7)	No negative APSQ with a positive GAD-7 [24,38] The best AUC value [8,14] AUC values: 0.84 [14], 0.89 [8], 0.92 [49] Good sensitivity (TPR > 80%) [46,49] Good specificity (>80%) [49] Low FPR [49]
Depressive symptoms (as measured by PHQ-9)	Good sensitivity (TPR > 80%) [46,49] Good specificity (>80%) [49] Good AUC values of 0.84 [14], 0.87 [8], and 0.91 [49] No negative APSQ with a positive PHQ-9 [24,38] Low FPR [49]
Self-harm (Q9 of PHQ-9)	Good sensitivity and specificity (>80%) [49] Good AUC values 0.82 [14], 0.86 [8], 0.90 [49] FNR/missed cases >0% [8,14] No FNR [46]
Sleep disturbances (as measured by ASSQ)	Low AUC values 0.69 [14], 0.67 [8], and 0.72 [49] High FNR/positive ASSQ with a negative APSQ (>15%) [46,48,49]
Alcohol misuse (as measured by AUDIT-C)	Low AUC 0.42 [14], 0.61 [8], and 0.64 [49] High FNR/missed cases (>18%) [8,38,46,49]
Drug use (as measured by CAGE-AID)	Low AUC value: 0.63 [14], 0.69 [49], acceptable AUC 0.74 [8] High FPR [46,49] High value of a negative APSQ with a positive CAGE-AID [49] Moderate value of missed cases (negative APSQ and positive CAGE-AID [38]) No negative APSQ with a positive CAGE-AID [24]
Disordered eating (as measured by BEDA-Q/EDE-QS)	AUC value 0.65 [8,14] Moderate accuracy for EDE-QS [49] Highest FNR [8,14] High FNR/negative APSQ with a positive BEDA-Q [24,38,46] Moderate FNR [49]
PTSD	Good TPR [46] High FPR [46]

## Data Availability

No new data were created or analysed in this study. Data sharing is not applicable to this article.

## Supplementary Materials

The following supporting information can be downloaded at: https://www.mdpi.com/article/10.3390/diagnostics16030486/s1. Supplementary Material Table S1: Chacarcteristics of the included studies. Supplementary Material Table S2: The Joanna Briggs Institute Critical Appraisal Checklist. File S1: Preferred Reporting Items for Systematic reviews and Meta-Analyses extension for Scoping Reviews (PRISMA-ScR) Checklist.

## Abbreviations

The following abbreviations are used in this manuscript:

AUDIT-C	Alcohol Use Disorders Identification Test Consumption
AUDIT	Alcohol Use Disorders Test
AUC	Area Under the Curve
APSQ	Athlete Psychological Strain Questionnaire
ASSQ	Athlete Sleep Satisfaction Questionnaire
BDSA	Baron Depression Screener for Athletes
BEDA-Q	Brief Eating Disorder in Athletes Questionnaire
CAGE-AID	Adjusted Form of the Cutting Down, Annoyance by Criticism, Guilty Feeling, and Eye-Openers Adapted to Include Drugs
χ^2^	Chi-Square Index
CFI	Comparative Fit Index
CR	Composite Reliability
CI	Confidence Interval
DASS-21	Depression, Anxiety, and Stress Scale
EDE-QS	Eating Disorder Examination—Questionnaire Short
GHQ-28	General Health Questionnaire
GAD-7	Generalised Anxiety Disorder Scale
IOC	International Olympic Committee
ICC	Intraclass correlation coefficient
KMO	Kaiser–Meyer–Olkin
K-10	Kessler Psychological Distress Scale 10
MHC-SF	Mental Health Continuum—Short Form
MHWG	Mental Health Working Group
MDC	Minimal Detectable Change
NFI	Normed Fit Index
PHQ-9	Patient Health Questionnaire-9 for Depression
PPV	Positive Predictive Value
RMSEA	Root Mean Square of Approximation
RMWBS	Ryff Mental Well-Being Scale
SMHAT-1	Sport Mental Health Assessment Tool-1
SEM	Standard Error Measurement
TLI	Tucker–Lewis Index
TPR	True Positive Rate
WEMWBS	Warwick–Edinburgh Mental Well-Being Scale
WHO-5	World Health Organisation Well-Being Index 5
YBOCS	Yale–Brown Obsessive–Compulsive Scale
