# Supplementary material for: International Validity of the Athlete Psychological Strain Questionnaire (APSQ): A Scoping Review
Source: Diagnostics (Basel). 2026 Feb 5;16(3):486. doi: 10.3390/diagnostics16030486 (PMC12896643; doi:10.3390/diagnostics16030486)
Supplement: Supplementary file 1 [file diagnostics-16-00486-s001.zip › diagnostics-4038978-supplementary.pdf]

Supplementary Material Table S1

Study	Type of Study	Number of Participants	Sample Characteristics	Aim	Limitations
**Alhowimel AS, et al., 2023 [37]**	Cross-sectional	98 athletes<br>21 athletes for the pretest<br>Elite +subelite	Inclusion criteria:<br>- Arabic speakers<br>- Ages between 18 and 45 years old<br>- Elite and Sub-elite athletes<br>- Consent<br>Exclusion criteria:<br>- Neurological condition<br>-Rheumatological condition	-Validation of the Arabic APSQ<br>- Internal consistency and test-retest reliability	-Male > Female<br>-Not a diverse sample in terms of socio-demographic aspects (such as gender, smoking status, marital status)<br>-Cultural influences – reluctance to reveal mental health difficulties<br>-Self-assessment design
**Ojio Y, et al., 2021 [3]**	Cross-sectional	612 male Japanese, elite rugby players only 227 consented to participate only **219** met the inclusion criteria; randomly divided calibration sample for exploratory factor analysis and a validation sample for confirmatory factor analysis	Inclusion criteria:<br>- Age ≥ 18 years old<br>- Athletes from the Japan Rugby Top League<br>-Japanese or international players	-Validation of the Japanese APSQ<br>-Comparison of the results with the ones reported for Australian athletes	-Exclusively male sample<br>-Low response rate<br>-Lack of diversity in terms of sport type<br>-Lack of test-retest reliability assessment<br>-Cultural influences: hesitancy to disclose mental health burdens<br>-80% of the athletes were not part of the national team<br>-No test-retest reliability testing<br>-Self-assessment design
**Lima Y, et al., 2022 [7]**	Cross-sectional	565 elite athletes - national team or top league<br>61 athletes - retested after 30 days	Inclusion criteria<br>- Ages between 18 and 38 years old<br>- Active participation all around the 2020-2021 season<br>- Top league or National team - elite athlete	-Test the reliability, validity and convergent validity of the Turkish APSQ	-Self-assessment design<br>-Overlap with the Covid pandemic<br>-Different demographic characteristics

			- Native Turkish speakers		(most of them single and young: 18 -22 y.o.)
**Azadi H, et. al, 2024 [19]**	Cross-sectional	317 athletes Elite and subelite	Inclusion criteria:<br>-Fluent in Persian<br>-Age > 16 years old<br>-Consent	-Explore the impact of the Covid pandemic on the mental health of athletes<br>-Validation of the Persian APSQ	-Fairly small number of participants<br>-Slightly imbalanced distribution - gender and type of sports (↑male ↑team sports)<br>-Cultural impact: tendency to hide mental health problems<br>-More than half of the participants were not part of the national team and did not compete in international competitions
**Sore K, et al., 2024 [36]**	Cross-sectional	869 elite athletes<br>20 athletes in the pilot study (translation process)	Inclusion criteria:<br>-16-65 years old<br>-sustained training (>10 h/week), 'professional' (competition in the last 52 weeks)<br><br>Exclusion criteria:<br>-incomplete responses<br>- more than 20% of missing replies	-Reliability and applicability of the Croatian APSQ	-Self-reported data<br>-Questionnaire distribution – managed by the clubs<br>-A proportion of variance - not correlated with the factors described<br>-Some factors might have been missed out
**Anderson T, et al., 2023 [14]**	Retrospective, observational	1066 USA athletes	Inclusion criteria:<br>-Athletes who competed at the Tokyo Olympic and Paralympic Games (2020) and Beijing Olympic and Paralympic Games (2022)	-Determine the false-negative rate of APSQ<br>-Determine the sensitivity and specificity of APSQ<br>-Explore if using APSQ together with all the questionnaires from the second	-Covid pandemic overlap<br>-Data collection during the Olympic Games<br>-Sample from only one country<br>-Q9 from the PHQ-9 might have

				step of APSQ is a better option	influenced the false negative rates<br>-There is not sufficient data to analyse the Paralympic athletes<br>-Sample size was not adequate for subgroup analysis, with post-hoc power analysis having statistical flaws<br>-Self-reported assessment
**Taylor DJ, et al., 2023 [48]**	Cross-sectional	993 student-athletes	Inclusion criteria:<br>Athletes from the University of Arizona	-Reliability assessment of the APSQ<br>-Cut-off scores assessment<br>-Comparison with the reported data from Australian athletes	-Self-reported assessment<br>-No validity testing against a clinical interview<br>-College athletes might not be representative of elite athletes<br>-Consent bias - those with fewer mental health issues might have a higher response rate<br>-Some questionnaires were not validated in athletes
**Ojio Y, et al., 2023 [38]**	Cross-sectional	220 male athletes - rugby	Inclusion criteria:<br>-Age ≥ 18 years old<br>-Japan Rugby Top League<br>-Consent<br>-Native Japanese<br>-Completed all the questions from the survey	Collect prevalence data	-Self-reported tool<br>-Solely male athletes<br>-Only one kind of sport<br>-Low response rate<br>-Did not test the reliability in time/for consecutive applications

**Rice SM, et al., 2020 [1]**	Cross-sectional	1007 elite Australian male athletes calibration sample - 497 athletes validation sample - 510 athletes	Inclusion criteria:<br>-Australian national team: football, soccer, cricket<br>-Age ≥ 18 years old	-Development of APSQ<br>-Validation of APSQ<br>-Determine convergent and divergent validity of APSQ against other validated tools<br>-Determine differences between injured athletes and uninjured athletes	-Solely male sample<br>-Did not assess test-retest reliability<br>-Further studies need to assess the correlation with mental health problems and sport-related variables, and the validity of the External Coping domain<br>-Self-assessment tool
**Waleriańczyk W, et al., 2024 [8]**	Cross-sectional	1121 professional athletes<br>576 males<br>545 females	Inclusion criteria:<br>Athletes who presented for the pre-participation examination at the National Centre for Sports Medicine<br>Consent	-Test the diagnostic performance of SMHAT-1 by benchmarking it against a clinical interview<br>-Determine the sensitivity and specificity of APSQ	-No data about the well-being<br>-During a clinical interview, athletes might conceal mental health problems<br>-A single-time measurement - does not offer information about the mental health dynamics
**Gouttebarge V, et. al, 2021 [24]**	Review and cross-sectional studies	In the cross-sectional study:<br>43 Sports Medicine Physicians<br>281 Professional Athletes	Inclusion criteria:<br>-Athletes ≥ 16 years old<br>-Australian<br>-Professional footballers<br>A- and W-League	Develop a mental health screening tool, adequate for athletes	-Self-reported tool<br>-Not all SMHAT-1 questionnaires were tested in the athletic population<br>-APSQ might overlook cases/give false alarms<br>-The sample did not include different types of sports or populations

					from different countries
**Shannon S, et al., 2024 [9]**	Cross-sectional	605 non-elite athletes<br>84 athletes in the retest survey -- within 1 month	Inclusion criteria:<br>-Consent<br>-Age ≥ 18 years old<br>-'Amateur' level athlete, according to a preliminary screening	Test the validity of the APSQ in an amateur athlete population	-The possibility of an inaccurate self-classification as a non-elite athlete<br>-Predominantly male athletes and those practising a team sport<br>-Specific stressors for non-elite athletes are not screened for in the APSQ<br>-Did not include Q9 from PHQ-9<br>-A small number of athletes in the retest phase<br>-An older population in the second phase of the study<br>-Self-reported tool
**Rice S, et al., 2020 [2]**	Cross-sectional	1093 elite athletes<br>1007 males<br>54 females	Inclusion criteria:<br>-Age ≥ 18 years old<br>-Elite athletes<br>-Australian athletes - part of the national team<br>-Australian football, cricket, soccer	-Determine the psychometric properties of APSQ: sensitivity, specificity, internal consistency, positive/negative correlations with other tools and validity in the context of injuries	-Self-assessment design<br>-The female sample was not sufficient to assess the correlations between injuries, gender and psychological strain
**Mountjoy M, et al., 2023 [15]**	Cross-sectional performed at three key moments	550 student-athletes<br>250 female athletes<br>300 male athletes<br>-At the first measurement: 542 athletes	Inclusion criteria:<br>-Student-athletes registered in the 2020-2021 academic year	-Determine the prevalence of mental health symptoms<br>-Assess the internal consistency of APSQ and step 2 of the Sport	-Did not differentiate the non-binary individuals<br>-The decreasing number of participants in the second and

		-At the second measurement: 336 athletes<br>-At the third measurement: 133 athletes		Mental Health Assessment Tool -1	third measurements<br>-Self-assessment tool<br>-The Covid pandemic influence
**García-Rubio J, et al., 2025 [43]**	Cross-sectional	128 athletes: amateur, sub-elite and elite athletes<br>71 male athletes<br>57 female athletes	<u>Inclusion criteria</u>:<br>-Age 18-65 years old<br>-Elite, sub-elite and amateur athletes<br>-Spanish knowledge<br>-Correct email address	-Test the validity of the APSQ Spanish version	-Small sample size<br><br>-Did not differentiate between the level of performance (elite vs amateur)<br><br>-Mostly amateur athletes<br><br>-Self-reported tool
**Anderson T, et. Al., 2025 [46]**	Secondary analysis	847 athletes: elite<br>621 Olympic athletes<br>226 Paralympic athletes	<u>Inclusion criteria</u>:<br>USA Olympic and Paralympic teams who participated at the Paris Olympic/Paralympic Games in 2024	-Test the diagnostic performance of the original APSQ	-Potentially biased sample - national teams<br>-Mixed administration method: online and in-person, with the in-person one not being anonymised - potential reluctance to disclose mental health problems

**Whelan BM, et al., 2026 [49]**	Secondary analysis - data collection between June 2020 and June 2023	2758 collegiate athletes (Division I)	Inclusion Criteria: Collegiate athletes from 5 Division I institutions	Determine the diagnostic performance of the original APSQ, alongside collecting prevalence data	-Potential unwillingness to disclose mental health problems<br>-Collegiate athletes might have specific characteristics, not applicable to the general athletic population<br>-Questionnaires used as benchmarks were not validated in collegiate athletes<br>-No data regarding the types of sports included<br>-Potentially unbalanced sample in terms of gender and ethnicity<br>-Cultural differences were not considered
**Yang J, et al., 2025 [47]**	Cross-sectional	1726 college USA athletes	Inclusion Criteria: Undergraduate and graduate students from USA Universities	-Test the APSQ validity in collegiate athletes and explore if there are differences in scores between genders.<br>-Explore correlations between the APS score and other tools/the average GPA	-The sample had characteristics that might not be applicable to the general population of athletes.<br>-Did not include gender minorities.<br>-Data collection during the Covid pandemic<br>-Possible reticence to disclose mental health issues.

**Putra, M.F.P., et al., 2025 [44]**	Cross-sectional	375 athletes: 241 male athletes and 134 female athletes	<u>Inclusion Criteria</u>:<br>Complete answers<br>Team and individual sports<br>Males and females<br>Those who participate in sports contests	-Test the validity of the Indonesian APSQ	-No EFA<br>-No other tools for correlation testing apart from the Mental Toughness Index<br>- Unequal distribution of the sample in terms of educational level, gender and sport type<br>-Significant multivariate kurtosis analysis

Supplementary Material Table S2 – The JBI Critical Appraisal Checklist for Cross-Sectional Studies

Moola, S.; Munn, Z.; Tufanaru, C.; Aromataris, E.; Sears, K.; Sfetcu, R.; Currie, M.; Lisy, K.; Qureshi, R.; Mattis, P.; Mu, P. Chapter 7: Systematic reviews of etiology and risk. Aromataris, E., Munn, Z., Eds.; In *Joanna Briggs Institute Reviewer's Manual*; The Joanna Briggs Institute: Adelaide, Australia, 2017.

**Study**	**Criteria 1**	**Criteria 2**	**Criteria 3**	**Criteria 4**	**Criteria 5**	**Criteria 6**	**Criteria 7**	**Criteria 8**
**Alhowimel AS, et al., 2023 [37]**	Yes	Yes	Not applicable	Self-reported tool	Not applicable to the validation analysis	Not applicable to the validation analysis	Self-reported tool	Yes
**Ojio Y, et al., 2021 [3]**	Yes	Yes	Not applicable	Self-reported tool	Not applicable to the validation analysis	Not applicable to the validation analysis	Self-reported tool	Yes
**Lima Y, et al., 2022 [7]**	Yes	Yes	Not applicable	Self-reported tool	Not applicable to the validation analysis	Not applicable to the validation analysis	Self-reported tool	Yes
**Azadi H, et. al, 2024 [19]**	Yes	Yes	Not applicable	Self-reported tool	Not applicable to the validation analysis	Not applicable to the validation analysis	Self-reported tool	Yes
**Sore K, et al., 2024 [36]**	Yes	Yes	Not applicable	Self-reported tool	Not applicable to the validation analysis	Not applicable to the validation analysis	Self-reported tool	Yes
**Anderson T, et al., 2023 [14]**	Yes	Yes	Not applicable	Self-reported tool	Not applicable to the validation analysis	Not applicable to the validation analysis	Self-reported tool	Yes
**Taylor DJ, et al., 2023 [48]**	Yes	Yes	Not applicable	Self-reported tool	Not applicable to the validation analysis	Not applicable to the validation analysis	Self-reported tool	Yes
**Ojio Y, et al., 2023 [38]**	Yes	Yes	Not applicable	Self-reported tool	Not applicable to the validation analysis	Not applicable to the validation analysis	Self-reported tool	Yes
**Rice SM, et al., 2020 [1]**	Yes	Yes	Not applicable	Self-reported tool	Not applicable to the validation analysis	Not applicable to the validation analysis	Self-reported tool	Yes

**Waleriańczyk W, et al., 2024 [8]**	Yes	Yes	Not applicable	Self-reported tool	Not applicable to the validation analysis	Not applicable to the validation analysis	Self-reported tool	Yes
**Gouttebarge V, et. al, 2021 [24]**	Yes	Yes	Not applicable	Self-reported tool	Not applicable to the validation analysis	Not applicable to the validation analysis	Self-reported tool	Yes
**Shannon S, et al., 2024 [9]**	Yes	Yes	Not applicable	Self-reported tool	Not applicable to the validation analysis	Not applicable to the validation analysis	Self-reported tool	Yes
**Rice S, et al., 2020 [2]**	Yes	Yes	Not applicable	Self-reported tool	Not applicable to the validation analysis	Not applicable to the validation analysis	Self-reported tool	Yes
**Mountjoy M, et al., 2023 [15]**	Yes	Yes	Not applicable	Self-reported tool	Not applicable to the validation analysis	Not applicable to the validation analysis	Self-reported tool	Yes
**García-Rubio J, et al., 2025 [43]**	Yes	Yes	Not applicable	Self-reported tool	Not applicable to the validation analysis	Not applicable to the validation analysis	Self-reported tool	Yes
**Anderson T, et. Al., 2025 [46]**	Yes	Yes	Not applicable	Self-reported tool	Not applicable to the validation analysis	Not applicable to the validation analysis	Self-reported tool	Yes
**Whelan BM, et al., 2026 [49]**	Yes	Yes	Not applicable	Self-reported tool	Not applicable to the validation analysis	Not applicable to the validation analysis	Self-reported tool	Yes
**Yang J, et al., 2025 [47]**	Yes	Yes	Not applicable	Self-reported tool	Not applicable to the validation analysis	Not applicable to the validation analysis	Self-reported tool	Yes
**Putra, M.F.P., et al., 2025 [44]**	Yes	Yes	Not applicable	Self-reported tool	Not applicable to the validation analysis	Not applicable to the validation analysis	Self-reported tool	Yes

File S1. Preferred Reporting Items for Systematic reviews and Meta-Analyses extension for Scoping Reviews (PRISMA-ScR) Checklist

SECTION	ITEM	PRISMA-ScR CHECKLIST ITEM	REPORTED ON PAGE #
**TITLE**			
Title	1	Identify the report as a scoping review.	1
**ABSTRACT**			
Structured summary	2	Provide a structured summary that includes (as applicable): background, objectives, eligibility criteria, sources of evidence, charting methods, results, and conclusions that relate to the review questions and objectives.	1
**INTRODUCTION**			
Rationale	3	Describe the rationale for the review in the context of what is already known. Explain why the review questions/objectives lend themselves to a scoping review approach.	3
Objectives	4	Provide an explicit statement of the questions and objectives being addressed with reference to their key elements (e.g., population or participants, concepts, and context) or other relevant key elements used to conceptualize the review questions and/or objectives.	3-4
**METHODS**			
Protocol and registration	5	Indicate whether a review protocol exists; state if and where it can be accessed (e.g., a Web address); and if available, provide registration information, including the registration number.	-
Eligibility criteria	6	Specify characteristics of the sources of evidence used as eligibility criteria (e.g., years considered, language, and publication status), and provide a rationale.	3-4
Information sources*	7	Describe all information sources in the search (e.g., databases with dates of coverage and contact with authors to identify additional sources), as well as the date the most recent search was executed.	4
Search	8	Present the full electronic search strategy for at least 1 database, including any limits used, such that it could be repeated.	5
Selection of sources of evidence†	9	State the process for selecting sources of evidence (i.e., screening and eligibility) included in the scoping review.	3-4
Data charting process‡	10	Describe the methods of charting data from the included sources of evidence (e.g., calibrated forms or forms that have been tested by the team before their use, and whether data charting was done independently or in duplicate) and any processes for obtaining and confirming data from investigators.	5-6
Data items	11	List and define all variables for which data were sought and any assumptions and simplifications made.	5-6
Critical appraisal of individual sources of evidence§	12	If done, provide a rationale for conducting a critical appraisal of included sources of evidence; describe the methods used and how this information was used in any data synthesis (if appropriate).	9
Synthesis of results	13	Describe the methods of handling and summarizing the data that were charted.	6

St. Michael's
Inspired Care.
Inspiring Science.

SECTION	ITEM	PRISMA-ScR CHECKLIST ITEM	REPORTED ON PAGE #
**RESULTS**			
Selection of sources of evidence	14	Give numbers of sources of evidence screened, assessed for eligibility, and included in the review, with reasons for exclusions at each stage, ideally using a flow diagram.	5
Characteristics of sources of evidence	15	For each source of evidence, present characteristics for which data were charted and provide the citations.	8-9 Plus Table S1
Critical appraisal within sources of evidence	16	If done, present data on critical appraisal of included sources of evidence (see item 12).	Table S2
Results of individual sources of evidence	17	For each included source of evidence, present the relevant data that were charted that relate to the review questions and objectives.	6-8; 9-12
Synthesis of results	18	Summarize and/or present the charting results as they relate to the review questions and objectives.	6-8;9-12
**DISCUSSION**			
Summary of evidence	19	Summarize the main results (including an overview of concepts, themes, and types of evidence available), link to the review questions and objectives, and consider the relevance to key groups.	12-14
Limitations	20	Discuss the limitations of the scoping review process.	13-14
Conclusions	21	Provide a general interpretation of the results with respect to the review questions and objectives, as well as potential implications and/or next steps.	14
**FUNDING**			
Funding	22	Describe sources of funding for the included sources of evidence, as well as sources of funding for the scoping review. Describe the role of the funders of the scoping review.	14

JBI = Joanna Briggs Institute; PRISMA-ScR = Preferred Reporting Items for Systematic reviews and Meta-Analyses extension for Scoping Reviews.

* Where *sources of evidence* (see second footnote) are compiled from, such as bibliographic databases, social media platforms, and Web sites.

† A more inclusive/heterogeneous term used to account for the different types of evidence or data sources (e.g., quantitative and/or qualitative research, expert opinion, and policy documents) that may be eligible in a scoping review as opposed to only studies. This is not to be confused with *information sources* (see first footnote).

‡ The frameworks by Arksey and O'Malley (6) and Levac and colleagues (7) and the JBI guidance (4, 5) refer to the process of data extraction in a scoping review as data charting.

§ The process of systematically examining research evidence to assess its validity, results, and relevance before using it to inform a decision. This term is used for items 12 and 19 instead of "risk of bias" (which is more applicable to systematic reviews of interventions) to include and acknowledge the various sources of evidence that may be used in a scoping review (e.g., quantitative and/or qualitative research, expert opinion, and policy document).

From: Tricco AC, Lillie E, Zarin W, O'Brien KK, Colquhoun H, Levac D, et al. PRISMA Extension for Scoping Reviews (PRISMAScR): Checklist and Explanation. Ann Intern Med. 2018;169:467–473. doi: 10.7326/M18-0850.
